# Radiation Physics and Safety in Fluoroscopy: A Clinician’s Guide to Principles and Practice

**DOI:** 10.7759/cureus.87245

**Published:** 2025-07-03

**Authors:** Kevin Rivera, Sam S Ahn

**Affiliations:** 1 Emergency Medicine, The Ohio State University Wexner Medical Center, Columbus, USA; 2 Surgery, Texas Christian University, Fort Worth, USA

**Keywords:** fluoroscopy, ionizing radiation, medical physics, occupational hazards, radiation safety

## Abstract

Fluoroscopic venography serves as a pivotal diagnostic and interventional modality in the assessment and management of venous disorders, including deep vein thrombosis, venous occlusion or malformations, and stent placement. The technique's real-time imaging capabilities facilitate precise visualization of venous anatomy and pathology, thereby guiding therapeutic interventions. However, the utilization of ionizing radiation inherent in fluoroscopy introduces potential risks to both patients and healthcare personnel. These risks encompass deterministic effects, such as skin erythema and cataract formation, and stochastic effects, including an increased likelihood of malignancy, particularly with cumulative exposure.

Despite the critical importance of radiation safety, many clinicians involved in fluoroscopic procedures lack comprehensive training in radiation physics and protective measures. This knowledge gap can lead to suboptimal practices, resulting in unnecessary radiation exposure and associated health risks. A thorough understanding of the fundamental principles of radiation physics, including the mechanisms of X-ray production, interactions with human tissue, and the quantification of radiation dose, is essential for optimizing patient care and ensuring occupational safety.

This review article aims to bridge the gap between theoretical radiation physics and its practical application in clinical settings. By elucidating the core concepts of radiation interactions, dose measurement, and safety protocols, we seek to empower clinicians with the knowledge necessary to minimize radiation exposure while maintaining diagnostic efficacy. Emphasis is placed on the implementation of the ALARA (As Low As Reasonably Achievable) principle, adherence to established dose limits, and the adoption of evidence-based strategies to mitigate radiation risks.

Enhancing radiation literacy among clinicians is paramount in the era of advanced imaging. By integrating foundational physics with clinical practice, healthcare providers can make informed decisions that prioritize both patient outcomes and occupational health.

## Introduction and background

Fluoroscopic venography has emerged as an indispensable tool in the diagnosis and management of various venous disorders, including deep vein thrombosis, venous occlusion or malformations, and stent placement. Its ability to provide dynamic, real-time imaging of venous structures allows for accurate assessment and targeted interventions, thereby improving patient outcomes. However, the benefits of fluoroscopy are accompanied by the inherent risks associated with ionizing radiation exposure, which can have significant implications for both patients and healthcare providers.

Ionizing radiation refers to high-energy electromagnetic waves or particles capable of removing tightly bound electrons from atoms, thereby creating charged ions. In fluoroscopy, X-rays constitute the primary form of ionizing radiation. These X-rays are generated within an X-ray tube when rapidly accelerated electrons strike a metal target. Two types of X-ray radiation are produced during this process: bremsstrahlung radiation (a continuous energy spectrum resulting from the deceleration of electrons near the nucleus) and characteristic X-rays (a discrete energy spectrum resulting from electron transitions between atomic orbitals within the target material) [1,2].

Once produced, X-rays interact with human tissue primarily via two mechanisms: the photoelectric effect and Compton scattering. The photoelectric effect involves complete absorption of an X-ray photon by an atom, leading to ejection of an inner-shell electron; this interaction contributes to image contrast but also increases patient dose. Compton scattering, more prevalent at higher photon energies, results in partial photon deflection and loss of energy, contributing to degraded image quality and occupational exposure [3,4].

Radiation exposure carries biological risks that are broadly categorized into deterministic and stochastic effects. Deterministic effects, such as skin injuries and cataract formation, have threshold doses and become more severe with increasing exposure. Stochastic effects, including carcinogenesis, have no known threshold and increase in probability with cumulative radiation exposure, even at low doses [5].

Radiation dose is quantified using multiple metrics. Absorbed dose, measured in grays (Gy), represents the energy deposited per unit mass of tissue. Equivalent dose, measured in sieverts (Sv), adjusts for the type of radiation using a radiation weighting factor. Effective dose, also expressed in sieverts, further accounts for tissue-specific sensitivity through a tissue weighting factor. Thus, equivalent dose reflects the type of radiation, while effective dose integrates both radiation type and biological sensitivity [6]. Typical dose ranges vary by procedure: a chest X-ray delivers approximately 0.1 mSv, while a CT scan of the abdomen and pelvis can deliver 10-20 mSv, depending on scanning parameters such as the use of contrast [7]. Fluoroscopic venography often results in doses between these values, generally increasing for prolonged or complex procedures.

Regulatory guidelines provide dose limits to protect occupational workers. The International Commission on Radiological Protection (ICRP) recommends a whole-body effective dose limit of 20 mSv per year, averaged over five years, with no single year exceeding 50 mSv. The annual limit for the lens of the eye has been lowered from 150 mSv in 2011 to 20 mSv due to growing evidence linking lower doses to cataract formation [8]. Cumulative exposure refers to the total amount of radiation a person receives over time from multiple procedures or occupational exposure. This accumulation is particularly relevant for healthcare workers in interventional settings, where small but frequent doses can lead to long-term health risks [9]. Pulsed fluoroscopy, a technique that emits X-rays in short bursts rather than continuously, is a key method for dose reduction. It decreases overall radiation exposure while maintaining sufficient image quality for diagnostic accuracy, particularly when lower pulse rates are used in conjunction with other protective strategies [10].

Improving radiation safety requires an integrated understanding of physics, dose metrics, and protection protocols. This review presents foundational principles in an accessible manner to support clinicians in minimizing radiation risks and fostering safer procedural environments.

## Review

Methods

This article is structured as a narrative educational review. Literature was selected through targeted searches of PubMed, ICRP publications, and international radiation safety guidelines from 2000 to 2025. Priority was given to studies, review articles, and regulatory documents relevant to fluoroscopy, venography, occupational exposure, and educational best practices in radiation safety. Due to the nature of the review, formal inclusion/exclusion criteria and a Preferred Reporting Items for Systematic Reviews and Meta-Analyses (PRISMA) flowchart were not applied.

Radiation physics and dose concepts for clinicians

The application of ionizing radiation in fluoroscopic venography requires a foundational understanding of the physical processes that govern both image formation and biological risk. These principles are not merely theoretical; they form the bridge between quantum physics and the clinical practice of imaging-guided interventions. A clinician’s ability to interpret fluoroscopy settings, appreciate tissue response, and mitigate radiation-related harm depends on their grasp of these concepts. Ionizing radiation refers to radiation energetic enough to displace electrons and ionize atoms, which is the fundamental principle behind imaging modalities that utilize X-rays. The photon, the basic unit of electromagnetic radiation, carries energy that determines the interaction behavior with tissue. Metrics such as absorbed dose, equivalent dose, and effective dose are critical for quantifying patient and operator exposure, guiding protective strategies [1,3,6]. To facilitate an understanding of the technical content presented in this review, Table 1 provides definitions of key radiation physics terminology with their clinical relevance.

**Table 1:** Essential definitions and units relevant to imaging-related radiation in clinical practice Adapted from references [2,8]

Term	Definition	Common Units	Clinical Relevance
Ionizing Radiation	Radiation with enough energy to remove tightly bound electrons from atoms, creating ions	N/A	Fundamental type of radiation used in imaging and procedures like venography.
Photon	A particle representing a quantum of light or other electromagnetic radiation	N/A	X-rays are high-energy photons used in diagnostic imaging.
Gray (Gy)	SI unit of absorbed dose-energy deposited per unit mass of tissue	J/kg	It measures how much radiation energy is absorbed by tissue.
Sievert (Sv)	Unit of equivalent/effective dose that accounts for the biological effects of radiation	J/kg (adjusted by weighting factors)	It is more clinically relevant than Gray for estimating stochastic risk (e.g., cancer).
Exposure	Measure of ionization in air due to X-rays or gamma rays	Coulomb/kg (C/kg)	It is used in radiology equipment calibration and environmental monitoring.
Scatter Radiation	Radiation deflected from its original path during interaction with matter	N/A	It increases the radiation dose and reduces image quality.
Effective Dose	Weighted sum of equivalent doses to all organs, accounting for sensitivity	Sievert (Sv)	It is used to estimate patient risk across imaging modalities.

The mechanism of X-ray generation involves two key processes: Bremsstrahlung radiation and characteristic X-ray production. Bremsstrahlung occurs when high-speed electrons decelerate upon encountering the atomic nucleus, emitting a continuous spectrum of X-ray photons. Characteristic radiation results from the rearrangement of inner-shell electrons following ionization events. These X-rays, when directed toward the patient, provide diagnostic information but also carry risk. Interaction with tissue primarily occurs through the photoelectric effect and Compton scattering. The photoelectric effect dominates at lower photon energies and in high atomic number materials such as iodinated contrast agents used in venography. Compton scattering prevails in soft tissue at higher energies and generates scattered photons that contribute to occupational dose and reduce image contrast [4,11].

From a practical standpoint, understanding dose parameters allows clinicians to compare imaging modalities and anticipate procedural risk. A single chest X-ray typically imparts around 0.1 mSv, while fluoroscopically guided venograms may deliver several millisieverts depending on duration, magnification, and field size. By recognizing these variations, clinicians can better weigh procedural necessity against biological cost [7,9]. Table 2 summarizes practical, evidence-based strategies that clinicians can adopt to reduce radiation exposure during fluoroscopic procedures.

**Table 2:** Practical strategies for radiation dose reduction in fluoroscopy Adapted from references [5,10,12,13] ALARA: As Low As Reasonably Achievable

Strategy	Description	Clinical Impact
Pulsed fluoroscopy	Emits radiation in bursts rather than continuously	Reduces patient and operator dose by 20–80% depending on pulse rate
Beam collimation	Narrows X-ray beam to the area of interest	Decreases patient exposure and improves image contrast
Low frame rate	Reduces the number of images per second (e.g., 15 vs. 30 fps)	Minimizes total dose without compromising diagnostic utility
Distance optimization	Maximizing operator distance from source	Applies inverse square law — doubling distance cuts dose by ~75%
Shielding	Lead aprons, thyroid collars, and ceiling-mounted shields	Primary defense against scatter radiation
Positioning	Stand on the image receptor side, minimize oblique angles	Reduces scatter direction toward operator
Real-time dose monitoring	Use of fluoroscopy timers and cumulative dose displays	Enhances situational awareness, promotes ALARA adherence

Radiation safety and occupational exposure in venography

Minimizing radiation exposure during fluoroscopic venography requires a comprehensive, team-based approach that incorporates both technology and behavior. Protective strategies fall into several categories: procedural technique, equipment design, shielding, and personnel practices.

Procedural techniques that reduce exposure include minimizing fluoroscopy time, using the lowest reasonable frame rate, avoiding unnecessary magnification, and employing collimation to restrict the X-ray beam to the area of interest. Pulsed fluoroscopy should be used whenever possible, as it emits radiation in intermittent bursts rather than continuously. This lowers the cumulative dose without substantially affecting image quality [10,12]. The choice of beam angulation can also impact scatter patterns. Shallow angles reduce operator exposure by redirecting scatter away from personnel [13].

Equipment setup and positioning play a crucial role in exposure mitigation. Fluoroscopy units equipped with dose-saving technology, such as automatic exposure control (AEC), spectral filtration, and last-image hold, should be properly utilized. When using a C-arm, detectors should be placed on the image-receiving side (above the patient) with the X-ray tube positioned below to direct scatter away from operators. Operators should maximize their distance from the beam whenever possible and stand on the detector side, leveraging the inverse square law to reduce dose [5,14].

Personal protective equipment (PPE) is essential and includes lead aprons, thyroid shields, and leaded eyewear. Aprons should be regularly inspected for cracks or wear. Ceiling-mounted shields and under-table drapes further limit scatter radiation and are most effective when used in combination. Studies have shown that omitting these tools leads to significantly higher operator doses [15]. Dosimeters are vital for monitoring occupational exposure by quantifying data to evaluate cumulative dose and compliance with safety thresholds. They should be worn at the collar outside the apron and, if available, under the apron as well. Proper use facilitates longitudinal tracking and prompts review of procedural practices when thresholds approach regulatory limits [16]. Institutions should provide training on interpreting dosimeter reports and responding to elevated readings.

Embedding the ALARA (As Low As Reasonably Achievable) principle into the workflow is essential for sustainable radiation safety. ALARA is not only a regulatory mandate but a philosophy of proactive exposure minimization. This includes fostering a culture where staff feel empowered to pause procedures when safety concerns arise and are trained to adjust imaging parameters without compromising diagnostic objectives. Institutions should hold regular training sessions, audit fluoroscopy usage, and display dose reports transparently within departments to reinforce accountability [17].

By integrating equipment knowledge, procedural awareness, and institutional support, radiation safety becomes both feasible and routine. These strategies collectively protect clinicians, technologists, and patients, allowing fluoroscopy to remain a powerful and safe tool in modern medicine.

Discussion

Fluoroscopic venography highlights a recurring challenge in modern medicine: balancing the clinical utility of advanced imaging with its biological consequences. While fluoroscopy enables precision in diagnosing and managing various disorders, it introduces invisible and cumulative risks to both patients and medical personnel. These risks, often underestimated or overlooked in real time, deserve active attention at every procedural stage.

This review has emphasized that radiation exposure is not evenly distributed. It varies by anatomy, technique, and operator position. Skin dose accumulates at the beam entry site. Eye dose increases with poor shielding. Staff exposure depends heavily on procedure duration, beam configuration, and protective behavior. In many cases, fluoroscopy settings are adjusted reflexively without full awareness of these gradients. Education around these factors can empower clinicians to tailor image acquisition to clinical goals without incurring unnecessary risk.

Fluoroscopy’s seamless integration into procedural workflows can obscure its consequences. Unlike a medication with a dosage label or lab value with trend lines, radiation lacks immediate feedback unless explicitly monitored. Without visual, tactile, or auditory cues, operators must rely on dose reports, timers, and fluoroscopic intuition. Incorporating tools like real-time dose displays, cumulative timers, and annotated images with dose metrics can bridge this cognitive gap and help cultivate situational awareness. Radiation protection reflects clinical culture and institutional values. The growing recognition of cataract risk, brain tumor incidence, and other occupational effects among interventionalists underscores the need for proactive change. Protective equipment must be seen as essential, not optional. As one example, dosimeter use should be routine, not reserved for audits. Leadership should support ongoing education, routine exposure tracking, and equipment upgrades.

This article advocates a more deliberate integration of radiation literacy into clinical education. Just as clinicians are taught to titrate vasopressors or interpret imaging artifacts, they must also understand how a change in kilovoltage affects contrast and dose. Concepts like effective dose, tissue weighting, and scatter geometry are safety levers that should shape procedural decisions. Interdisciplinary training sessions, simulation environments, and inclusion of real dose metrics during teaching cases can foster this fluency.

Ultimately, embedding radiation safety into daily practice strengthens clinical effectiveness while keeping both patients and clinicians safe. Recognizing and applying these principles transforms radiation safety from a compliance task to a clinical competency, and one that balances image quality with biological cost. Radiation is not just an imaging tool; it is a measurable, modifiable exposure that demands informed stewardship.

Limitations

This narrative educational review aimed to synthesize essential radiation physics and safety principles relevant to fluoroscopic venography. However, it does not follow a formal systematic review framework. No PRISMA diagram or structured search strategy was applied, and the literature base was selectively drawn from major guidelines, foundational texts, and key studies. Consequently, there is a risk of selection bias, and the comprehensiveness of the literature base may be limited. Additionally, although practical recommendations are emphasized, quantitative modeling or procedural dose audits were beyond the scope of this article.

## Conclusions

Fluoroscopic venography stands at the intersection of clinical necessity and radiation responsibility. While its clinical value in managing venous disease is well-established, its reliance on ionizing radiation carries significant implications for both patient and clinician safety. This review re-examines the physics that govern fluoroscopy to empower clinicians with the knowledge to make safer, more informed decisions at the bedside and in the interventional suite. Understanding radiation generation, tissue interactions, and dose quantification enables proceduralists to actively minimize harm without compromising efficacy. This may involve adjusting beam parameters, collaborating more effectively with technologists, or modeling best practices for learners and peers.

A cultural and systemic shift in radiation safety is urgently needed. Protection must move beyond regulatory compliance to become a core tenet of clinical professionalism. Occupational exposure is not incidental. It is cumulative, measurable, and preventable. Through the consistent use of personal protective equipment, adherence to the ALARA principle, real-time dosimetry, and institutional commitment to safety culture, the risks of fluoroscopy can be substantially mitigated. As procedural volume and complexity continue to rise, so too must our fluency in the unseen forces that shape our imaging decisions. Radiation is not simply a tool; it is a responsibility. Future work should focus on embedding this knowledge into training, reinforcing it through leadership, and tracking it with data to ensure that the invisible risks of radiation are matched with visible accountability.

## References

[1] Frane N, Bitterman A (2023). Radiation safety and protection. StatPearls [Internet].

[2] Smith FA (2000). A Primer in Applied Radiation Physics.

[3] Johnson MM (2015). Radiation protection education in fluoroscopy. Radiol Technol.

[4] Young HD, Freedman RA, Ford AL (2007). University Physics. 12th edn.

[5] Schueler BA (2003). Personnel protection during fluoroscopic procedures. AAPM Annual Meeting. AAPM Annual Meeting.

[6] International Commission on Radiological Protection (2019). Dose limits. International Commission on Radiological Protection. https://icrpaedia.org/Dose_limits.

[7] Mettler FA Jr, Huda W, Yoshizumi TT, Mahesh M (2008). Effective doses in radiology and diagnostic nuclear medicine: a catalog. Radiology.

[8] ICRP ICRP (2007). The 2007 recommendations of the International Commission on Radiological Protection. Ann ICRP.

[9] Lin EC (2010). Radiation risk from medical imaging. Mayo Clin Proc.

[10] ICRP ICRP (2004). Managing patient dose in digital radiology. ICRP Publication 93. Ann ICRP.

[11] Bushber JT, Seibert JA, Leidholdt EM Jr, John M. Boone JM (2021). The Essential Physics of Medical Imaging. Edition 4. Med Phys.

[12] Vano E, Kleiman NJ, Duran A, Romano-Miller M, Rehani MM (2013). Radiation-associated lens opacities in catheterization personnel: results of a survey and direct assessments. J Vasc Interv Radiol.

[13] Dauer LT, Thornton RH, Miller DL (2012). Radiation management for interventions using fluoroscopic or computed tomographic guidance during pregnancy: a joint guideline of the Society of Interventional Radiology and the Cardiovascular and Interventional Radiological Society of Europe with Endorsement by the Canadian Interventional Radiology Association. J Vasc Interv Radiol.

[14] (2010). National Council on Radiation Protection and Measurements. NCRP Report No. 168. Radiation dose management for fluoroscopically-guided interventional medical procedures. National Council on Radiation Protection and Measurements.

[15] Vano E, Gonzalez L, Fernández JM, Haskal ZJ (2008). Eye lens exposure to radiation in interventional suites: caution is warranted. Radiology.

[16] Kim KP, Miller DL, Balter S, Kleinerman RA, Linet MS, Kwon D, Simon SL (2008). Occupational radiation doses to operators performing cardiac catheterization procedures. Health Phys.

[17] International Atomic Energy Agency (2018). International Atomic Energy Agency. Occupational radiation protection. https://www.iaea.org/publications/11113/occupational-radiation-protection.

